# Cten Is Targeted by Kras Signalling to Regulate Cell Motility in the Colon and Pancreas

**DOI:** 10.1371/journal.pone.0020919

**Published:** 2011-06-16

**Authors:** Saleh Al-Ghamdi, Abdulkader Albasri, Julien Cachat, Salih Ibrahem, Belal A. Muhammad, Darryl Jackson, Abdolrahman S. Nateri, Karin B. Kindle, Mohammad Ilyas

**Affiliations:** 1 Division of Pathology, Nottingham University, Nottingham, United Kingdom; 2 Division of Pre-Clinical Oncology, Nottingham University, Nottingham, United Kingdom; 3 Nottingham Digestive Diseases Centre, NIHR Biomedical Research Unit, Queen's Medical Centre, Nottingham University Hospitals NHS Trust, Nottingham, United Kingdom; University of Birmingham, United Kingdom

## Abstract

*CTEN/TNS4* is an oncogene in colorectal cancer (CRC) which enhances cell motility although the mechanism of Cten regulation is unknown. We found an association between high Cten expression and *KRAS/BRAF* mutation in a series of CRC cell lines (p = 0.03) and hypothesised that Kras may regulate Cten. To test this, Kras was knocked-down (using small interfering (si)RNA) in CRC cell lines SW620 and DLD1 (high Cten expressors and mutant for *KRAS*). In each cell line, Kras knockdown was mirrored by down-regulation of Cten Since Kras signals through Braf, we tested the effect of Kras knockdown in CRC cell line Colo205 (which shows high Cten expression and is mutant for *BRAF* but wild type for *KRAS*). Cten levels were unaffected by Kras knockdown whilst Braf knockdown resulted in reduced Cten expression suggesting that Kras signals via Braf to regulate Cten. Quantification of Cten mRNA and protein analysis following proteasome inhibition suggested that regulation was of Cten transcription. Kras knockdown inhibited cell motility. To test whether this could be mediated through Cten, SW620 cells were co-transfected with Kras specific siRNAs and a Cten expression vector. Restoring Cten expression was able to restore cell motility despite Kras knockdown (transwell migration and wounding assay, p<0.001 for both). Since *KRAS* is mutated in many cancers, we investigated whether this relationship could be demonstrated in other tumour models. The experiments were repeated in the pancreatic cancer cell lines Colo357 & PSN-1(both high Cten expressors and mutant for *KRAS*). In both cell lines, Kras was shown to regulate Cten and forced expression of Cten was able to rescue loss of cell motility following Kras knockdown in PSN-1 (transwell migration assay, p<0.001). We conclude that, in the colon and pancreas, Cten is a downstream target of Kras and may be a mechanism through which Kras regulates of cell motility.

## Introduction

C-terminal tensin-like (*Cten*, *TNS4*) is a member of the *Tensin* gene family. This gene family comprises four members (*TNS1, TNS2, TNS3 and TNS4/CTEN*) and their products are localised to the cytoplasmic tails of integrins at focal adhesions. Tensins play an important role in various biological processes such as cell adhesion, migration, proliferation, differentiation, apoptosis and invasion [1], [2], [3]. Human tensins 1, 2 and 3 are highly homologous at their N- and C-termini, but tensin4 / COOH-terminus Tensin-like molecule (Cten) is a smaller protein which shows C-terminus homology but does not contain the N-terminus actin-binding domain that is present in the other tensin proteins [4].

The role of Cten in neoplasia is complex. Mutations have not been described and changes in wild type Cten expression seem to be context dependent – it is down-regulated in prostate cancer [4] and is therefore thought to act as a tumour suppressor. In contrast , it is up-regulated and therefore acts as an oncogene in many cancers including breast cancer and colorectal cancer (CRC) [5], [6], [7]. We have recently shown that high levels of Cten expression are associated with a poor prognosis in breast cancer [8] and similar results have been reported in thymomas, gastric cancers and lung cancers [9], [10], [11]. The impact of Cten expression on clinical outcome may be related to its biological activity which, in colorectal cancer cell lines at least, results in enhanced colony formation, resistance to staurosporine-induced apoptotic stress and increased cell motility (both cell migration and cell invasion) [5], [7].

Tensin family proteins interact with several structural and signalling molecules such as vinculin, paxillin, Src, Focal Adhesion Kinase (FAK), phosphatidylinositol-3-kinase (PI3-K), and Crk-associated substrate p130^CAS^, actin as well as integrins. Since Cten is a recently described gene, data about either its regulation or its downstream targets are sparse. In breast cancer, Cten is positively regulated by c-Erb-B2 protein [6] – this is over-expressed in a specific subset of breast cancers due to gene amplification and is a part of the Epidermal Growth Factor Receptor (EGFR) signalling pathway. The EGFR pathway signals through Kras/Braf and although EGFR/c-Erb-B2 amplifications are rare in CRC, gain-of-function mutations in *KRAS/BRAF* are extremely common and are seen in up to 60% of tumours [12], [13]. We have previously studied a series of CRC cell lines for both expression of Cten and somatic mutation in a several known oncogenes / tumour suppressors [5], [13]. Combined evaluation of these data showed a significant association between high Cten expression and *KRAS/BRAF* mutation (p = 0.03, Table 1 and Table S1). This led us to hypothesise that Cten is a target of Kras/Braf signalling and in this study we sought to test this hypothesis and thereby elucidate the mechanisms of Cten regulation. We tested our hypothesis in CRC cell lines and then validated the findings in pancreatic cancer cell lines. The latter were chosen to represent a different type of cancer which also has a high frequency of *KRAS* mutation.

**Table 1:** Association of Cten expression Kras/Braf mutation.

	Kras/Braf status of cell line	
	Mutant	Wild Type
**Low Cten expression**	4	6
**High Cten expression**	15	3

A series of cell lines had previously been analysed for Cten expression and Kras/Braf mutation. Analysis of the data showed a significant association between up-regulation of Cten expression (i.e. greater than normal mucosa) and mutation in any of the Kras/Braf hotspots (Fisher's exact test, p = 0.03).

## Materials and Methods

### Tissue culture

The CRC cell lines used in this study (SW620, DLD-1, Colo 205, RKO) were kindly donated by Prof I Tomlinson and have been previously described [5], [13]. The pancreatic cell lines, Colo357 and PSN-1 are well described cell lines [14], [15] which both contain mutant *KRAS*. All cell lines were cultured in Dulbecco's modified Eagle's medium (DMEM; Invitrogen) supplemented with 10% fetal calf serum (Invitrogen) and 1% penicillin/streptomycin (Invitrogen) in 5% CO_2_ in a humidified atmosphere.

### Transfection of cell lines and proteasomal inhibition

In order to knock down Cten, Braf and Kras, cells were transfected with small interfering RNA (siRNA) duplexes using Lipofectamine 2000 (Invitrogen) as previously described [16]. The cells were transfected with each siRNA duplex at a final concentration of 100 nM and compared with cells transfected with sequence scrambled control (ssc) duplexes (i.e. duplexes with the same base composition arranged in a random order). Two different duplexes were used for Kras knockdown and the sequences of the gene-specific duplexes and scrambled controls are as follows: (1) Kras specific 5′CAGGGUGUUGAUGAUGCCUUCUAUA′3 and scrambled control 5′CAGUGUAGUAGUCGUUUCUCGGAUA′3 (2) Kras specific 5′UAUAGAAGGCAUCAUCAACACCCUG′3 and scrambled control 5′UAUCCGAGAAACGACUACUACACUG′3.

In order to induce Cten expression, cells were transfected with a CMV promoter driven expression construct containing Green Fluorescent Protein (GFP) tagged Cten (GFP-Cten) using Lipofectamine 2000 (Invitrogen) as previously described [5]. Control cells were transfected with a GFP empty vector. In order to inhibit Wnt signalling, cells were transfected with an expression vector containing dominant negative (DN) TCF4 as previously described [17].

In order to functionally evaluate the functional relationship between Kras and Cten, a specific cellular condition was created whereby Kras protein was depleted and Cten protein was restored. This was achieved by co-transfection of Kras-specific siRNA and GFP-Cten and, for this purpose, the protocol for siRNA transfection was used with appropriate amounts of plasmid mixed in with the siRNA prior to adding to Lipofectamine 2000. Cells were analysed 72 hours later by Western blot or functional assays.

In order to ascertain the role of proteasomal degradation in the Kras mediated regulation of Cten, the proteasomal inhibitor Z-Leu-Leu-Phe-CHO, (Sigma , USA), was added to SW620 cells 24 hours after transfection with siRNA duplexes (either Kras specific or scrambled control).

### RNA extraction and Quantitative RT-PCR (Q-RT-PCR)

Total RNA was extracted from cells using the RNeasy Mini Kit (Qiagen) following the manufacturer's protocols and quantified on a NanoDrop ND-1000 UV-Vis Spectrophotometer (LabTech International Ltd, Ringmer, UK as previously described [16]. Complementary DNA (cDNA) was synthesized using M-MLV reverse transcriptase (Invitrogen) in accordance with the manufacturer's instructions.

Quantification of Cten and Kras was performed using the standard curve method. All experiments were conducted in triplicate and test gene values were normalized to the housekeeping gene *HPRT*. Each PCR was of a final volume of 25 µl and contained 10 ng of cDNA template, 1× SYBR Green Master Mix (Stratagene) and 250 nM primers. PCR was performed on an MX3005P Real-time PCR machine (Stratagene, UK) and cycling conditions were 5 minutes denaturation at 95°C followed by 40 cycles of: 30 seconds denaturation at 95°C/30 seconds annealing [60°C for *Cten*/59°C for *HPRT*/50°C for Kras/30 seconds extension at 72°C and a single final extension for 10 minutes. The data for Q-PCR were analyzed using the MxPro-QPCR software for Mx3005P QPCR system. PCR primer sequences are available from the authors.

### Western blotting

Whole cell extracts were prepared using lysis buffer (20 mM Tris, pH 7.5, 150 mM NaCl, 1% TritonX-100, 0.5% sodium deoxycholate, 1 mM EDTA, 0.1% SDS, supplemented with protease and phosphatase inhibitors (Sigma)). 30 µg protein was loaded on a 10% SDS–PAGE gel and transferred onto PVDF membranes by semi-dry transfer. After blocking, membranes were incubated overnight at room temperature with the indicated primary antibody. Antibodies were used at the following dilution: anti-Cten (Sigma, WH0084951M1, 1∶1000), anti-E-Cadherin (Abcam, ab1416, 1∶1000), mouse anti-Kras (Abcam, ab16795, 1∶250), mouse anti-β-actin (Sigma, 1∶2000) and mouse anti-CD24 (SWA11 – supernatant specific for an N-terminal epitope, a kind gift from Prof Altevogt). After three washes in TBS/Tween-20 (0.05%), blots were incubated for 1 hour at room temperature with the appropriate horseradish peroxidase-linked secondary antibody. After three further washes, detection was performed using the Enhanced Chemiluminescence Kit (Pierce). Bands were visualised using X-Ray films (Kodak) and quantified using ImageJ software.

### Flow cytometry

In order to test the transfection efficiency of GFP-Cten into SW620, cells were initially transfected with GFP-CTEN as above. After 48 hours, the cells were non-enzymatically detached by incubating with TE buffer (10 mM Tris-HCl (pH 8), 1 mM EDTA, Sigma) for 5–10 minutes. These then underwent anlaysis in an Epics Altra Flow cytometer (Beckman Coulter). 600,000 events were analysed using a GFP filter with the appropriate gate parameter.

Confirmation of the efficacy of the DN-TCF expression vector was tested by the effect on CD24 expression (a well known Wnt target gene) [18], [19]. Forty eight hours after transfection with the DN-TCF construct, cells were non-enzymatically detached and approximately 5×10^5^ cells were incubated with PE-labelled anti-CD24 antibody (BD Biosciences) diluted 1∶100 in FACS wash (0.5% bovine serum albumin; 2 mM NaN_3_; 5 mM EDTA) for 15 minutes at 4°C. An isotype and concentration matched PE labelled control antibody (Miltenyi Biotec, UK) was used and samples labelled with this antibody were used to set the gating levels. After three 5 minute washes with FACS wash, the cells were re-suspended and fixed in solution containing FACS wash with 2% formaldehyde. Determination of percentage of CD24+ cells was performed on an Epics Altra flow cytometry machine (Beckman Coulter). The results were analyzed using WinMDi 2.9 computer software.

### Cell Migration/ Invasion Assays

Transwell cell migration was measured using a Boyden chamber containing a polycarbonate filter with an 8 µm pore size (Costar). Five ×10^4^ cells were seeded and cell migration was assessed after 48 hr by fixing the cells attached to the lower surface in 10% methanol for 30 minutes. These were then stained for 30 minutes with methylene blue and the stained cells manually counted. Cell invasion was measured in the same way as described for migration, except that prior to cell seeding the upper chamber was prepared by coating the filter with 100 µl Matrigel (5 mg/ml; BD Biosciences) and the cells attached to the lower surface were fixed, stained and counted. Cell wounding migration assays were performed in six-well plates. Cells were grown to confluence and then serum-deprived for 24 hrs. A sterile 200 µl pipette tip was used to create three separate parallel wounds and migration of the cells across the wound line was assessed at 0, 24, 48 and 72 hr. Photographs were taken using a charge-coupled device (CCD) camera (Canon, Japan) attached to the inverted phase-contrast microscope at a power of 40×. In order to statistically analyse cell migration in the wounding assay, images were taken from three separate wounds All images were taken at the same magnification and converted into binary images using the ImageJ image analysis program (http://rsb.info.nih.gov/ij/index.html) and the area of the wound was measured. All assays were performed in triplicate on at least two separate occasions.

### Statistical analysis

Statistical analysis was undertaken using SPSS 13. 0 Software. All evaluations were done using unpaired two tailed Student's T-test. For cell counting studies cell numbers (as quantified by the methylene blue assay) were analysed. For the cell wounding studies, total pixel numbers with an intensity value of 0 were analysed as previously described [5]. A two-tailed p-value of <0.05 was considered statistically significant.

## Results

### (a) Cten and cell motility

**Figure 1:**
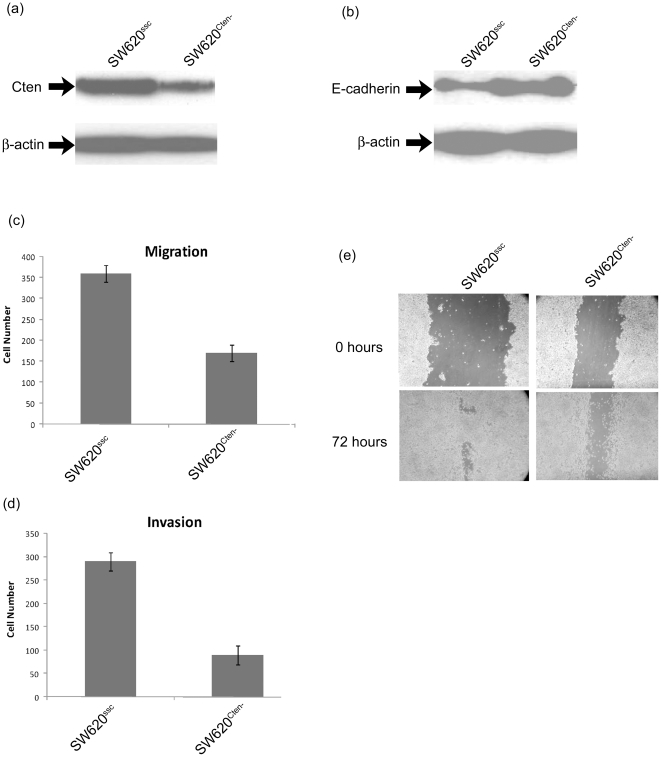
Cten stimulates cell motility and alters E-cadherin levels. (a) We have previously shown that forced expression of Cten induces cell motility. In the reverse experiment, we knocked down Cten protein expression with Cten specific siRNAs (SW620^Cten-^) compared with scrambled controls (SW620^ssc^). (b) Knockdown of Cten was associated with up-regulation of E-cadherin. (c) Functionally, knockdown of Cten caused reduction in transwell migration (upper panel, p<0.001) and transwell invasion through matrigel (lower panel, p<0.001). (d) Wound healing assays shown delayed closure of wound following Cten knockdown (p<0.001). Upper panel shows wound at time 0 hours and lower panel shows the same field after 72 hours.

We have previously shown that forced expression of Cten induces cell motility in cell lines not expressing Cten [5]. In order to validate these data, we conducted the reverse experiment of Cten knockdown in SW620. This is a CRC cell line showing high Cten expression (Figure 1a). The knockdown of Cten was also associated with up-regulation of E-cadherin (Figure 1b) thereby validating our previous observation of E-caderin down-regulation following ectopic expression of Cten. SW620 cells transfected with Cten specific siRNAs showed a reduction in transwell migration and transwell invasion through matrigel (p<0.001 for both, Figure 1c and d) when compared with cells transfected with scrambled controls. Similarly, wound healing assays showed delayed closure of wound following Cten knockdown (p<0.001, Figure 1e).

### (b) Kras and Cten expression

**Figure 2:**
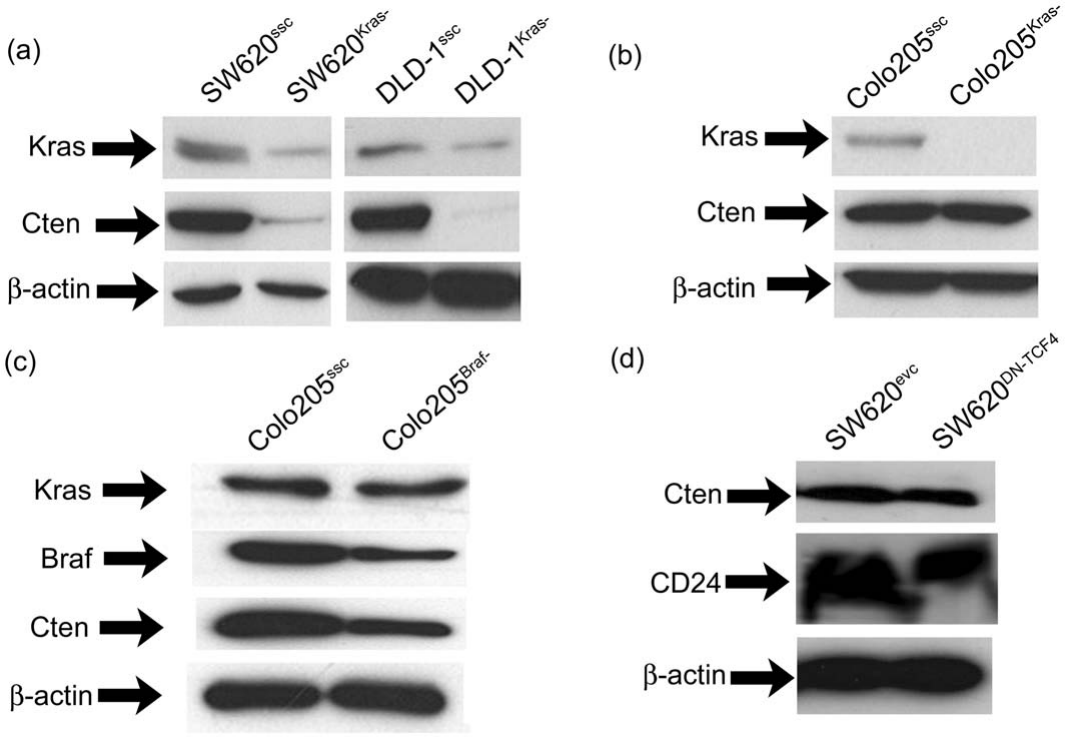
Functional relationship between Kras and Cten. (a) Knockdown of Kras in SW620 and DLD1 (both mutant for *KRAS*) resulted in a down-regulation of Cten levels (SW620^Kras-^ versus SW620^ssc-^, DLD1^Kras-^ versus DLD1^ssc-^). In contrast, (b) shows that Kras knockdown in Colo205 (containing a *BRAF* V600E mutation) had no effect on Cten expression (Colo205^Kras-^ versus Colo205^ssc^) whilst (c) shows that knockdown of Braf in Colo205 did cause down-regulation of Cten (Colo205^Braf-^ versus Colo205^ssc^). Figure (d) shows that inhibition of Wnt signalling in SW620 with a dominant-negative TCF4 expression vector (SW620^DN-TCF4^) did not alter Cten levels compared with empty vector (SW620^evc^) whilst levels of CD24 (a known target of Wnt signalling) were reduced.

Having found an association between Cten expression and *KRAS/BRAF* mutation in a series of CRC cell lines (Table 1), we sought to examine whether these were functionally linked. Firstly, Kras was knocked down in SW620. This cell line contains a *KRAS* mutation and is a high expressor of Cten. Knockdown of Kras in SW620 (annotated as SW620^Kras-^) resulted in down-regulation of Cten (Figure 2a) when compared with scrambled controls (SW620^ssc^). This effect was validated in the cell line DLD1 which also contains a *KRAS* mutation and is a high expressor of Cten (DLD1^Kras-^ versus DLD1^ssc^) both of which produced Kras knockdown and both of which resulted in down-regulation of Cten.

Kras signals through Braf and, in order to further test the association between Kras and Cten, Kras was knocked down in the cell line Colo205 (containing a mutation in *BRAF* but wild type for *KRAS*). In this case the level of Cten was unaffected (Colo205^Kras-^ versus Colo205^ssc^, Figure 2b). However when Braf was knocked down in Colo205, a 52% reduction in Braf protein (as evaluated by densitometry) was mirrored by a 50% reduction in Cten expression (Colo205^Braf-^ versus Colo205^ssc^, Figure 2c) suggesting that Kras influences Cten through Braf.

Since there is early up-regulation of Cten i.e. during the adenomatous phase of tumour development, the possibility that it may be a target of Wnt signalling was tested. CD24 is a well described target of Wnt signalling [18], [19] and dominant-negative TCF4 was transfected into SW620. Quantitative Western blot demonstrated a 40% reduction in the expression of CD24 and 53% reduction in cells expressing CD24 by flow cytometry (Figure S1). However transfection of DN-TCF4 into SW620 did not influence Cten expression (Figure 2d) suggesting that Wnt signalling does not play a role in regulating Cten expression.

### (c) Kras regulates Cten transcription

**Figure 3:**
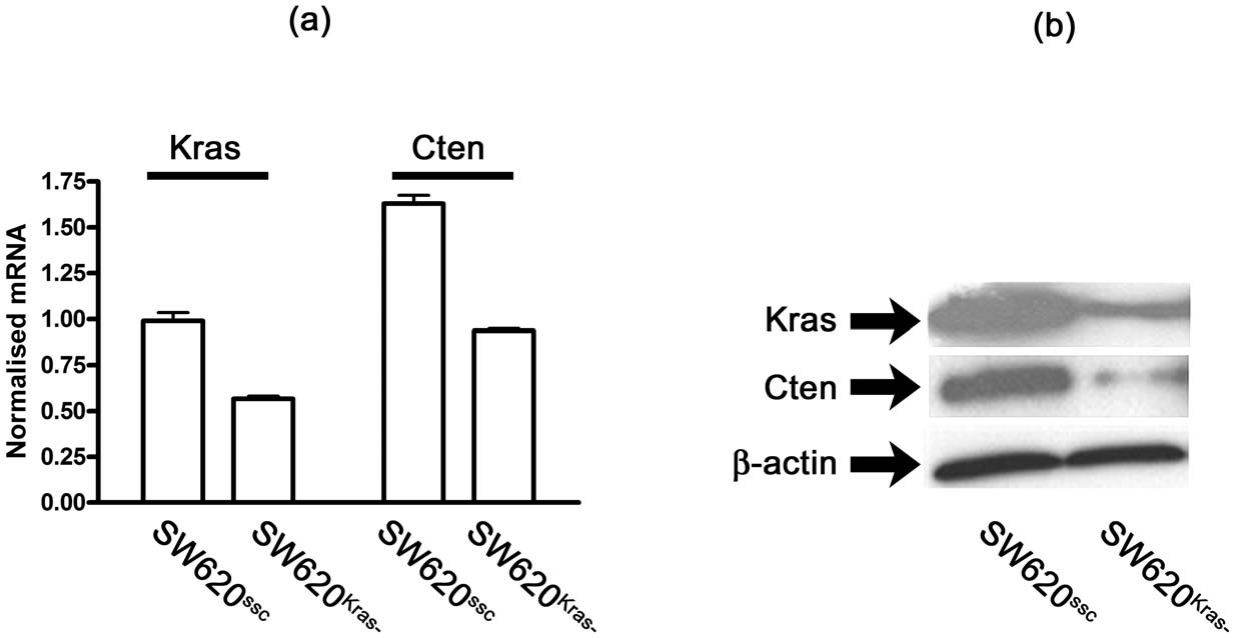
Kras regulates Cten through transcriptional control. (a) Following Kras knockdown in SW620, mRNA levels of both Kras and Cten were quantified and normalised to the housekeeping gene HPRT. There was a reduction in the level of both mRNAs (data shown from three replicates). (b) To further support this, cells were exposed to a proteasome inhibitor and levels of Kras and Cten proteins quantified. There was a reduction in levels of both proteins suggesting that the changes in Cten levels following Kras knockdown were not due to altered degradation. Controls consisting of DMSO carrier alone had no affect on protein levels (data not shown).

Our data showed that Cten is regulated, in part at least, by Kras/Braf signalling although it was uncertain whether this occurs through direct up-regulation of transcripton or through inhibition of degradation. In order to test this, Cten mRNA levels were measured by quantitative RT-PCR following Kras knockdown. This showed that there was a 43% reduction in normalised Cten mRNA levels (Figure 3a) suggesting there was transcriptional regulation. To further validate this, cells were exposed to a proteasomal inhibitor following Kras knockdown. This would be expected to prevent protein degradation but it did not prevent reduction in the levels of Cten (Figure 3b, Figure S2) thus supporting the quantitative PCR results which suggested that Kras/Braf signalling alters the transcription of Cten to regulate Cten protein levels.

### (d) Functional interaction between Kras and Cten

**Figure 4:**
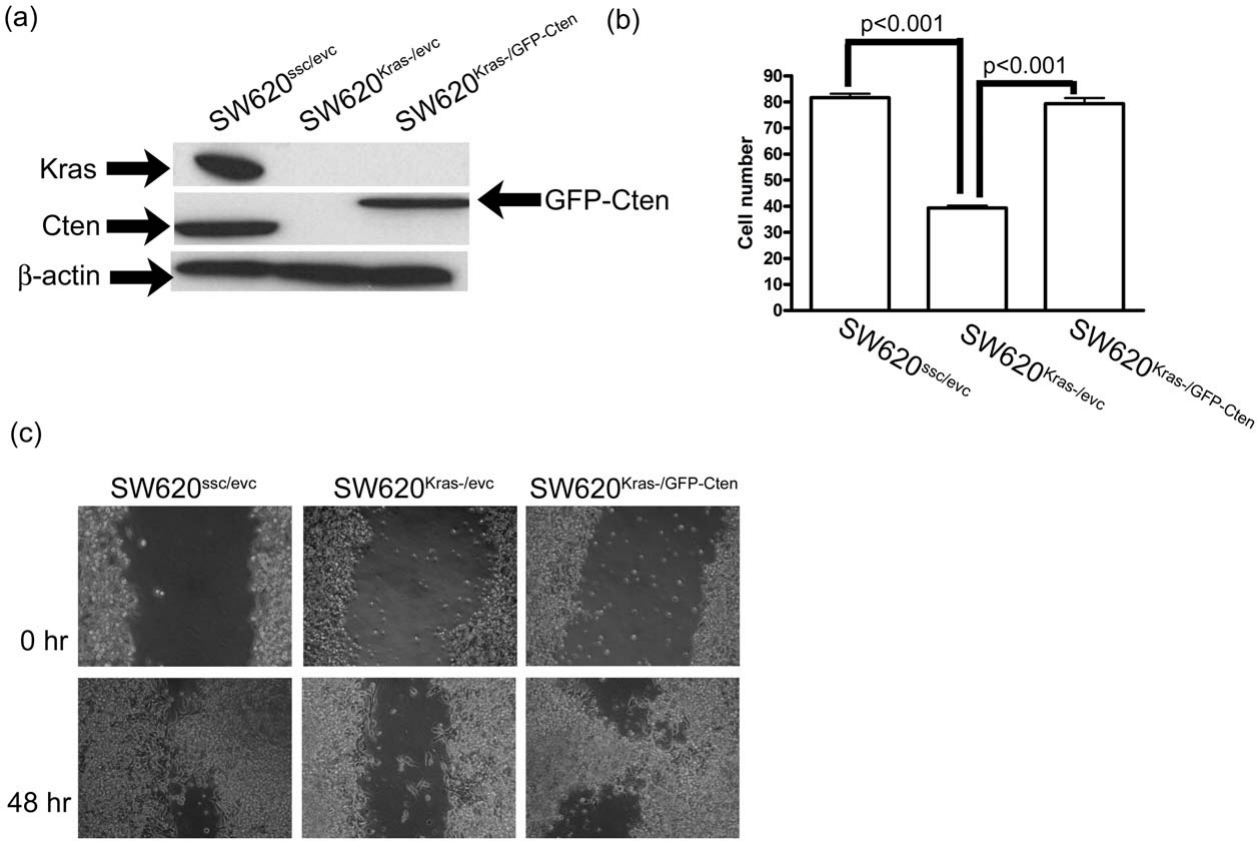
The relationship between Kras and Cten in the colon. (a) SW620 cells were transfected with Kras-specific siRNA and cells were either co-transfected with GFP-Cten expression vector (SW620^Kras-/GFP-Cten^) to restore Cten or GFP empty vector (SW620^Kras-/evc^). Control cells were co-transfected with scrambled controls siRNA duplexes and GFP empty vector (SW620^ssc/evc^). The ectopically expressed Cten has a larger size due to the GFP tag. (b) Transwell migration assays and (c) wounding assays showing that in SW620, knockdown of Kras inhibited cell motility which was rescued by the ectopic expression of Cten. (EVC = empty vector control, GFP = Green Fluorescent Protein, SSC = sequence scrambled controls).

Kras was shown to positively regulate Cten but, since Kras has several hundred downstream targets, it is possible that this is a reproducible observation without any direct functional relevance to cell biology. In order to test whether this relationship had any functional effect, we compared the conditions of Kras knockdown with Kras knockdown and concomitant ectopically expressed Cten protein. SW620 cells were thus co-transfected with Kras-specific siRNA duplexes and a construct causing ectopic expression of Green Fluorescent Protein (GFP) tagged Cten (SW620^Kras-/GFP-Cten^) and compared with cells co-transfected with Kras specific siRNA and GFP expressing empty vector controls (SW620^Kras-/evc^). Experimental controls were cells co-transfected with scrambled siRNA duplexes and GFP empty vector (SW620^ssc/evc^) creating a condition in which neither Kras nor Cten were altered (Figure 4a). Although creation of the appropriate conditions was confirmed by Western blotting, for the purposes of cell motility studies, it was necessary to confirm a high efficiency of transfection of the GFP-Cten expression vector into the cells. Flow cytometry demonstrated that a transfection efficiency of 60% was achieved (Figure S3).

Knockdown of Kras with co-transfection of GFP empty vector significantly reduced cell motility in transwell migration assay (Figure 4b, SW620^ssc/evc^ versus SW620^Kras-/evc^, p<0.001). However, ectopic expression of GFP-Cten restored the cell motility (SW620^Kras-/evc^ versus SW620^Kras-/GFP-Cten^, p<0.001). Cell wounding assays using the same transfection protocol demonstrated the same effect and validated the data (Figure 4c, p<0.001). Thus the data show that the effect of Kras knockdown on cell motility can be rescued by ectopic expression of Cten thereby confirming the functional nature of the relationship between Kras and Cten.

### (e) Kras/Cten in pancreatic cancer

**Figure 5:**
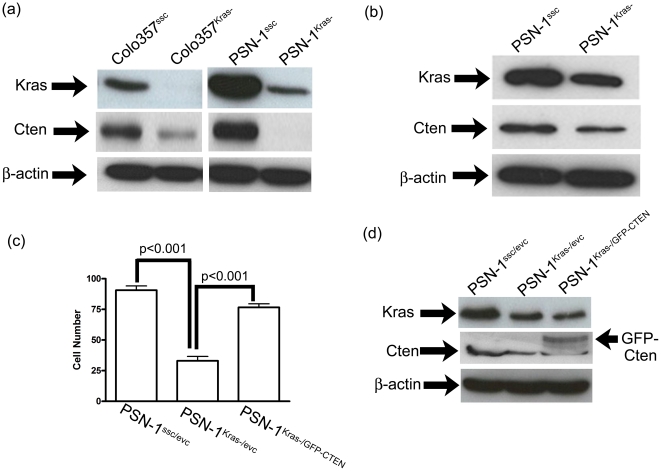
The relationship between Kras and Cten in the pancreas. Experiments to test the relationship between Kras and Cten were repeated in pancreatic cancer cell lines. (a) shows that when Kras was knocked down in Colo357 and PSN1, this caused down-regulation of Cten (Colo357^Kras-^ versus Colo357^ssc-^, PSN-1^Kras-^ versus PSN-1^ssc-^). In order to obviate any confounding off-target effects, a second anti-Kras siRNA duplex was used. (b) shows that the second Kras specific duplex also resulted in reduction in both Kras levels and Cten levels. (c) shows that, as observed in the colon, in pancreatic cell lines, knockdown of Kras inhibited motility (PSN-1^Kras-/evc^ versus PSN-1^ssc/evc^) and this could rescued by transfection of GFP-tagged Cten (PSN-1^Kras-/evc^ versus PSN-1^Kras-/GFP-Cten^). (d) is confirmation of the changes induced by the gene knockdown/forced expression in the rescue experiments.

Our current data have shown that, in CRC, Kras appears to regulate Cten and, through this, to regulate cell motility. In order to test whether this was a colon-specific relationship or whether it also occurred in other tumour types, the experiments were repeated in cell lines derived from pancreatic cancers. This was chosen as a model because pancreatic cancer has a high frequency of *KRAS* mutation. Colo357 and PSN1 are both pancreatic cancer cell lines which show high Cten expression and are reportedly mutant for *KRAS*. Knockdown of Kras in both cell lines resulted in down-regulation of Cten (Figure 5a) compared with scrambled controls. A second siRNA duplex targeted to Kras was also tested to preclude “off-target” effects and this also showed inhibition of Cten expression (Figure 5b) . Functional studies to further test this relationship were performed in PSN1 and once again co-transfections were used to create the conditions whereby Kras alone was knocked down (PSN1^Kras-/evc^), Kras was knocked and Cten restored (PSN1^Kras-/GFP-Cten^) and neither was altered (PSN1^ssc/evc^). The data paralleled those in the CRC cell lines and Kras knockdown was demonstrated to inhibit cell motility (PSN1^Kras-/evc^ versus PSN1^ssc/evc^, p<0.001) whilst this could be rescued by restoration of Cten expression (PSN1^Kras-/evc^ versus PSN1^Kras-/GFP-Cten^, p<0.001, Figure 5c).

## Discussion

Our previous study, in which we had forcibly expressed Cten protein in cell lines which were negative for Cten expression, suggested that Cten is involved in the regulation of cell migration and invasion. Although this was demonstrated in two different cell lines, we felt it necessary to confirm these observations using a different technique. Knockdown of Cten in the CRC cell line SW620 (which expresses high levels of Cten) repressed cell motility and caused up-regulation of E-cadherin thereby validating our previous data and firmly establishing the role of Cten in regulating cell motility.

Little is known of the mechanism of Cten regulation and, in this study, we have found an association between high Cten expression and *KRAS/BRAF* mutation in CRC cell lines. We have demonstrated Cten is a true target of Kras signalling since (i) knockdown of Kras results in down-regulation of Cten in two cell lines which are mutant for *KRAS* and (ii) knockdown of Kras in a cell line mutant for *BRAF* has no effect on Cten expression whilst knockdown of Braf in this cell line does result in down-regulation of Cten. Furthermore, quantification of Cten mRNA and use of proteasomal inhibitors to prevent protein degradation suggested that the level of control lay at Cten transcription.

Taking cognizance of the fact that there are a large number of reported targets of Kras and it is unlikely that they will all be functionally relevant [20], we have shown that the relationship between Kras and Cten is functionally important since inhibition of motility following Kras knockdown can be rescued by ectopic expression of Cten. Furthermore, we have demonstrated that the interaction between Kras and Cten is similar in pancreatic cancer suggesting that this is a generic relationship which is not limited to CRC.

This is the first report of Cten as a target of Kras signalling although it is clear that there are other mechanisms controlling Cten expression since we have identified occasional cell lines mutant for *KRAS* which show low levels of Cten. Conversely, there are cell lines which are wild type for *KRAS/BRAF* but which have high levels of Cten. Recently Stat3 has been reported as a modulator of Cten expression [21] and may represent an alternate pathway for Cten regulation. Currently, we can speculate that when Cten expression is elevated in a tumour with *KRAS/BRAF* mutation, the association is likely to be causal.

Our data suggest that a Kras-Cten signalling pathway exists which regulates cell motility. Taken together with studies in breast cancer showing that EGFR signalling can regulate Cten expression, a wider EGFR-Kras-Cten signalling pathway can be inferred. Circumstantial support for this comes from studies showing high levels of Cten expression in lung cancer [10]; tumours in this organ have a high frequency of disrupted EGFR/Kras signalling due to either Kras or EGFR mutation. However, Kras acts as a secondary messenger for a large number of Receptor Tyrosine Kinases (RTKs) in addition to EGFR and many of these, on ligand binding, can stimulate cell motility [22]. It is possible, albeit speculative at this stage, that Cten may represent a mechanistic link between many different RTKs and remodelling focal adhesions which will likely be an essential part of the process of inducing cell motility. Furthermore, our demonstration that E-cadherin levels are modulated by Cten provides a potential means of cross-talk and co-ordination between integrin and cadherin mediated cell adhesion.

As well as raising some fascinating questions about the role of Cten in regulating cell motility, our data also have some therapeutic implications. The use of biologics targeted to the EGFR can have a dramatic effect on CRC resulting in marked reduction in tumour size and clinical down-staging [23]. However, as would be expected, tumours containing *KRAS/BRAF* mutation are refractory to the therapeutic effects of anti-EGFR antibodies. If future studies confirm that Cten is a part of the EGFR-Kras signalling pathway, it may represent a new therapeutic option for the significant number of CRCs (approximately 60%) containing *KRAS/BRAF* mutation and therefore ineligible for anti-EGFR therapy.

## Supporting Information

Figure S1 Validation of the functional activity of DN-TCF4 was shown by flow cytometry. The expression of cell surface CD24 was tested following transfection into SW620 of either an expression construct expressing DN-TCF4 (SW620^DN-TCF4^) or empty vector (SW620^evc^). Gating levels were ascertained using an isotype PE-labelled control antibody and it was shown that DN-TCF4 resulted in a 53% reduction in cells expressing CD24.
(TIF)

Figure S2 To confirm that the proteasomal inhibitor (PI) was effective, RKO cells were incubated either with the inhibitor or with DMSO and levels of β-catenin were quantified. Exposure to the proteasomal inhibitor resulted in inhibition of β-catenin degradation making it detectable by Western blotting.
(TIF)

Figure S3 High efficiency of transfection of GFP-Cten into SW620 was confirmed by flow cytometry. To ensure that exposure to lipofectamine (the transfection reagent) did not influence fluorescence, control cells were transfected with a scrambled siRNA control. In comparison with control cells, there was approximately 60% transfection efficiency.
(TIF)

Table S1 This is a table of the cell lines showing fold change in *CTEN* message expression compared with the mean value for normal mucosa (as previously described in Albasri et al. J Pathol 2009; 218, 57–65). The column alongside indicates whether there is a mutation in the hotspots of KRAS or BRAF.
(DOC)
