# Supplementary figures and images for: Cten Is Targeted by Kras Signalling to Regulate Cell Motility in the Colon and Pancreas
Source: PLoS One. 2011 Jun 16;6(6):e20919. doi: 10.1371/journal.pone.0020919 (PMC3116852; doi:10.1371/journal.pone.0020919)

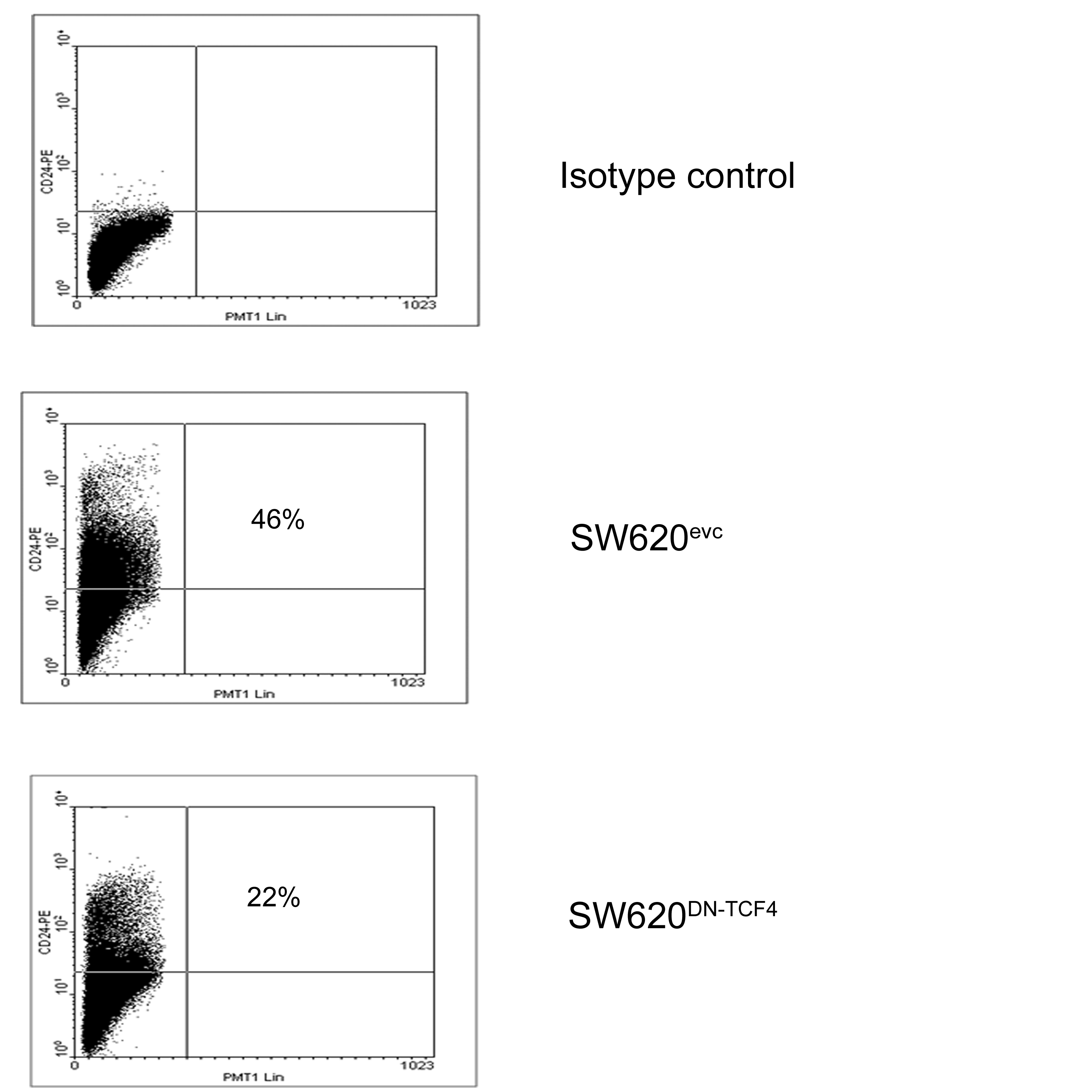

Supplement: Figure S1 — Validation of the functional activity of DN-TCF4 was shown by flow cytometry. The expression of cell surface CD24 was tested following transfection into SW620 of either an expression construct expressing DN-TCF4 (SW620DN-TCF4) or empty vector (SW620evc). Gating levels were ascertained using an isotype PE-labelled control antibody and it was shown that DN-TCF4 resulted in a 53% reduction in cells expressing CD24. (TIF) [file pone.0020919.s001.tif]

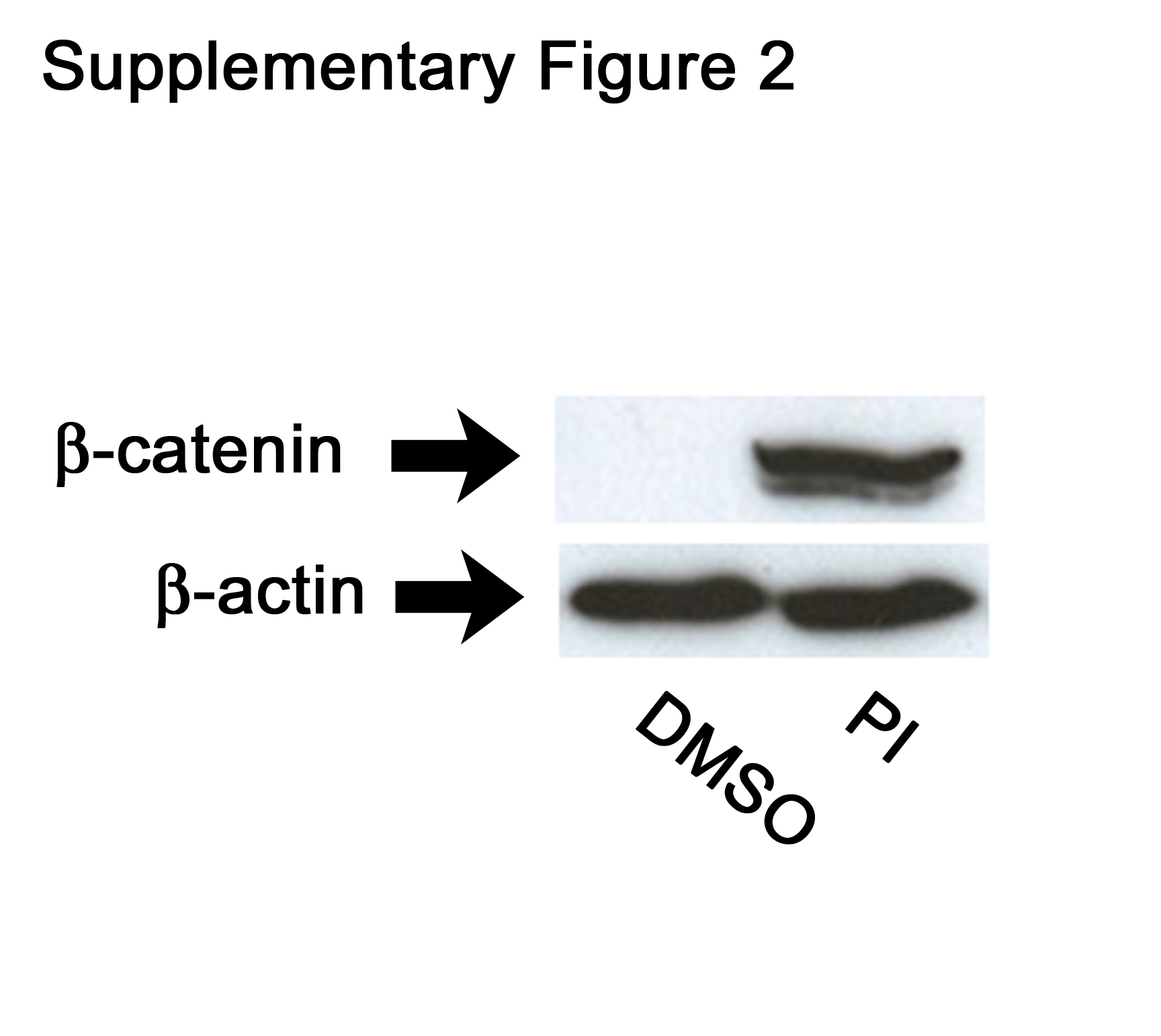

Supplement: Figure S2 — To confirm that the proteasomal inhibitor (PI) was effective, RKO cells were incubated either with the inhibitor or with DMSO and levels of β-catenin were quantified. Exposure to the proteasomal inhibitor resulted in inhibition of β-catenin degradation making it detectable by Western blotting. (TIF) [file pone.0020919.s002.tif]

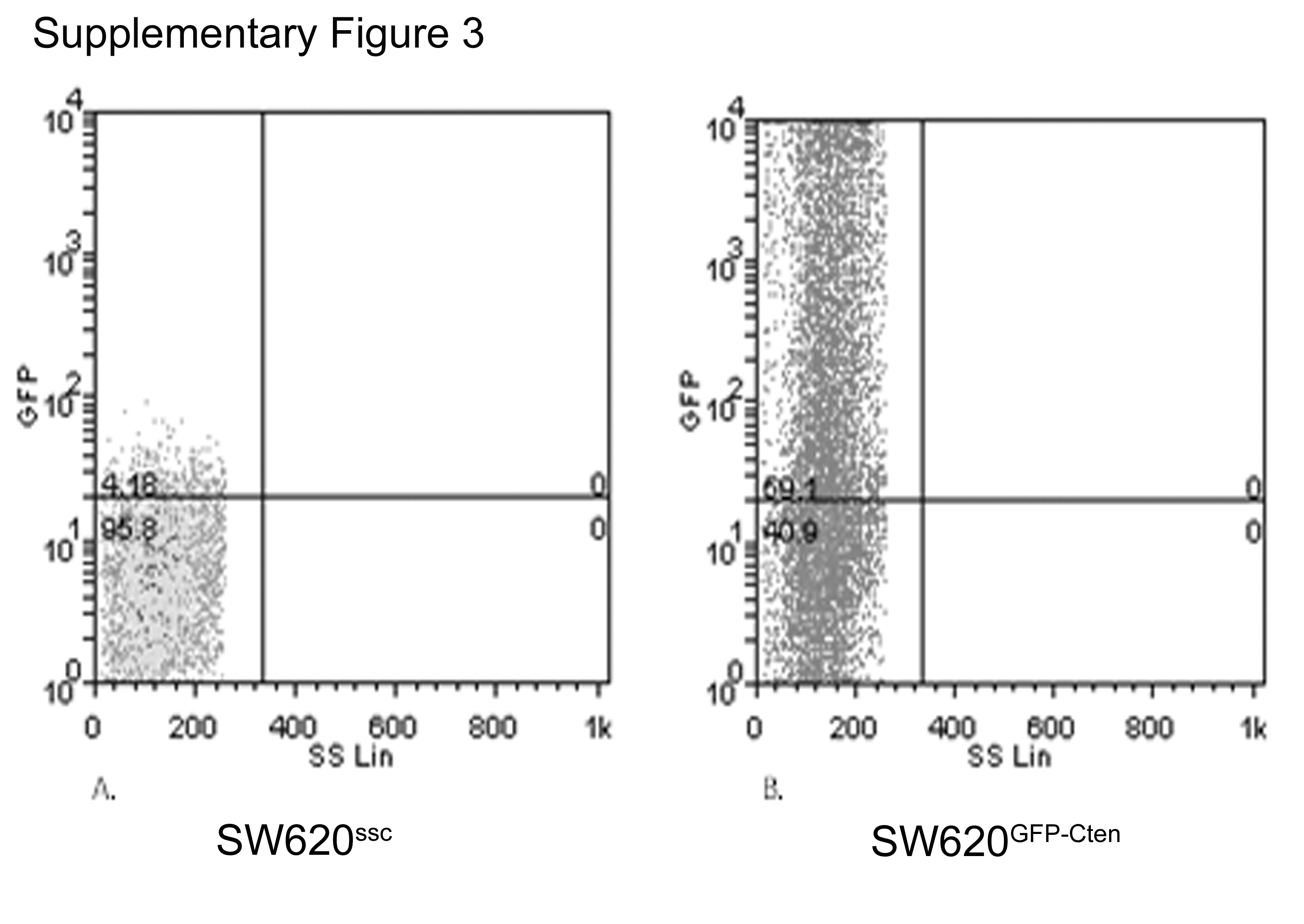

Supplement: Figure S3 — High efficiency of transfection of GFP-Cten into SW620 was confirmed by flow cytometry. To ensure that exposure to lipofectamine (the transfection reagent) did not influence fluorescence, control cells were transfected with a scrambled siRNA control. In comparison with control cells, there was approximately 60% transfection efficiency. (TIF) [file pone.0020919.s003.tif]
