# Supplementary material for: Cten Is Targeted by Kras Signalling to Regulate Cell Motility in the Colon and Pancreas
Source: PLoS One. 2011 Jun 16;6(6):e20919. doi: 10.1371/journal.pone.0020919 (PMC3116852; doi:10.1371/journal.pone.0020919)
Supplement: Table S1 — This is a table of the cell lines showing fold change in CTEN message expression compared with the mean value for normal mucosa (as previously described in Albasri et al. J Pathol 2009; 218, 57–65). The column alongside indicates whether there is a mutation in the hotspots of KRAS or BRAF. (DOC) [file pone.0020919.s004.doc]

**Supplementary Table 1 Expression of CTEN in Colorectal cancer cell lines compared with normal mucosa and association with KRAS/BRAF mutation**

| **Cell lines** | **Fold difference** | **KRAS/BRAF mutation** |
| --- | --- | --- |
| DLD1 | 57.1 | Yes |
| COLO205 | 51.4 | Yes |
| LOVO | 48.6 | Yes |
| SW620 | 48.6 | Yes |
| COLO201 | 35.7 | Yes |
| C80 | 7.1 | Yes |
| GP2D | 7.1 | Yes |
| VACO10MS | 7.1 | Yes |
| C32 | 4.3 | No |
| C84 | 4.3 | Yes |
| HCA46 | 4.3 | No |
| SW1116 | 4.3 | Yes |
| VACO5 | 4.3 | Yes |
| C125 | 3.6 | No |
| LS1034 | 3.6 | Yes |
| SW1222 | 3.6 | Yes |
| SW480 | 3.6 | Yes |
| SW948 | 2.9 | Yes |
|  |  |  |
| COLO320DM | 0.7 | No |
| HT29 | 0.7 | Yes |
| HT55 | 0.7 | No |
| RKO | 0.7 | Yes |
| HCA7 | 0.6 | No |
| HCT116 | 0.6 | Yes |
| HUTU80 | 0.6 | No |
| HRA19 | 0.3 | No |
| CACO2 | 0.2 | No |
| SW837 | 0.2 | Yes |
